# Intranasal Application of Budesonide Attenuates Lipopolysaccharide-Induced Acute Lung Injury by Suppressing Nucleotide-Binding Oligomerization Domain-Like Receptor Family, Pyrin Domain-Containing 3 Inflammasome Activation in Mice

**DOI:** 10.1155/2019/7264383

**Published:** 2019-02-27

**Authors:** Liang Dong, Yu-Hang Zhu, De-Xing Liu, Juan Li, Peng-Cheng Zhao, Yuan-Ping Zhong, Yong-Qin Chen, Wei Xu, Zhao-Qiong Zhu

**Affiliations:** Department of Anesthesiology, Affiliated Hospital of Zunyi Medical University, Zunyi, Guizhou 563000, China

## Abstract

**Aim:**

To investigate the protective effects of budesonide against lipopolysaccharide- (LPS-) induced acute lung injury (ALI) in a murine model and its underlying mechanism.

**Methods:**

Adult male C57BL/6 mice were divided into three groups: control, ALI, and ALI + budesonide groups. LPS (5 mg/kg) was intratracheally injected to induce ALI in mice. Budesonide (0.5 mg/kg) was intranasally given 1 h before LPS administration in the ALI + budesonide group. Twelve hours after LPS administration, all mice were sacrificed. Hematoxylin-eosin staining and pathological scores were used to evaluate pathological injury. Bronchoalveolar lavage was performed. The numbers of total cells, neutrophils, and macrophages in the bronchoalveolar lavage fluid (BALF) were counted. Enzyme-linked immunosorbent assay was employed to detect the proinflammatory cytokines in BALF and serum, including tumor necrosis factor- (TNF-) *α*, monocyte chemoattractant protein- (MCP-) 1, and interleukin- (IL-) 1*β*. The expression of the nucleotide-binding oligomerization domain-like receptor family, pyrin domain-containing 3 (NLRP3) inflammasome was detected by western blotting. A lethal dose of LPS (40 mg/kg, intraperitoneally) was injected to evaluate the effects of budesonide on survival rates.

**Results:**

Budesonide pretreatment dramatically attenuated pathological injury and reduced pathological scores in mice with ALI. Budesonide pretreatment obviously reduced the numbers of total cells, neutrophils, and macrophages in the BALF of mice with ALI. Additionally, budesonide dramatically reduced TNF-*α* and MCP-1 expression in the BALF and serum of mice with ALI. Budesonide significantly suppressed NLRP3 and pro-caspase-1 expression in the lung and reduced IL-1*β* content in the BALF, indicating that budesonide inhibited the activation of the NLRP3 inflammasome. Furthermore, we found that budesonide improved the survival rates of mice with ALI receiving a lethal dose of LPS.

**Conclusion:**

Suppression of NLRP3 inflammasome activation in mice via budesonide attenuated lung injury induced by LPS in mice with ALI.

## 1. Introduction

Acute respiratory distress syndrome (ARDS) is a devastating clinical condition with high mortality [1], characterized by uncontrolled inflammation, pulmonary edema, and decreased lung compliance [2]. Unfortunately, there are no specific pharmacological therapies for ARDS, only with supportive management [3]. Acute lung injury (ALI) was first described in 1967 and is mainly used in an experimental setting, because all experimental animal models fail to fulfill the complete Berlin definition [4, 5]. Intratracheal (*i.t.*) instillation of lipopolysaccharide (LPS), a bacterial cell wall component, is a common and well-accepted experimental model for ALI in mice [6].

Growing studies have shown that effective control of inflammation is the best treatment for ALI. Glucocorticoids are a powerful anti-inflammatory drug. However, systemic side effects, such as immunosuppression and infection, limit the clinical application of glucocorticoid [7]. Budesonide, an inhaled glucocorticoid, may maximize therapeutic benefits with fewer systemic side effects. Recently, a double-blind, randomized clinical trial indicated that early treatment with inhaled budesonide and beta agonist is feasible and improves oxygenation for patients with ARDS [8]. In addition, budesonide inhalation ameliorates endotoxemia-induced lung injury in rabbits [9], ventilator-induced lung injury in rats [10], and saline-lavage ALI in rabbits [11]. However, the effects of budesonide on LPS-induced ALI in mice and the underlying mechanisms remain unclear.

The inflammasome is a unique multiprotein complex that has been shown to play a central role in orchestrating the initiation and amplification of inflammatory responses [12]. Although inflammasome activation is critical for host defenses against microbial infections by inducing caspase-1 activation and interleukin- (IL-) 1*β* family maturation [13], excessive inflammasome activation induces tissue injury [14]. The nucleotide-binding oligomerization domain-like receptor (NLR) family, pyrin domain-containing 3 (NLRP3) inflammasome is the best studied inflammasome [15]. Our previous study demonstrated that inhibition of the NLRP3 inflammasome attenuates LPS-induced ALI in mice [16]. Similar results were found in other studies [17–19]. Interestingly, prednisone inhibits the NLRP3 inflammasome and reduces the release of cytokines, alleviating cuprizone-induced demyelination in a murine model [20]. Nuclear factor kappa B (NF-*κ*B) signaling contributes to NLRP3 inflammasome activation [19, 21], and budesonide can suppress NF-*κ*B [22].

In this study, we hypothesized that budesonide may attenuate LPS-induced ALI in mice *via* inhibition of the NLRP3 inflammasome. To test this, we used an LPS-induced murine ALI model to investigate the protective effects of budesonide against ALI.

## 2. Materials and Methods

### 2.1. Animal Model of ALI

All experimental protocols were performed in accordance with the ethical guidelines of the Ethics Committee of Zunyi Medical University. Adult male C57BL/6 mice were bred in the animal facility at Zunyi Medical University. All surgeries were performed under anesthesia with intraperitoneal injection of pentobarbital sodium (80 mg/kg). Mice were randomly divided into three groups: the control, the ALI, and budesonide + ALI groups (*n* = 8 each group). LPS (5 mg/kg, O111:B4 from *Escherichia coli*; Sigma-Aldrich, St. Louis, MO, USA) in 50 *μ*L sterile saline was intratracheally injected to establish the murine model of ALI. Mice in the control group received 50 *μ*L sterile saline only (*i.t.*). To study the effects of budesonide, mice with LPS-induced ALI were administered with intranasal administration of 50 *μ*L of budesonide (0.5 mg/kg) 1 h before LPS injection. This dose was chosen based on our own preliminary data and other previous studies [23, 24]. The mice were sacrificed at 12 h after LPS injection. Lung tissues were collected and snap-frozen at -80°C for future analysis. Lung tissues for pathological analysis were fixed with formalin.

### 2.2. Bronchoalveolar Lavage Fluid (BALF) Collection

Twelve hours after the administration of LPS, the thorax was opened, and ice-cold sterile saline (0.8 mL) was instilled into the lungs three times according to our previous study [25]. The samples were used only when the recovery rate was more than 95% [26]. Recovered BALF was centrifuged at 450 × *g* for 10 min. The cell-free supernatants were used for detection of protein concentrations or cytokine measurements. The cell pellets were resuspended in 0.5 mL PBS, and the number of neutrophils was counted with a hemocytometer and Wright-Giemsa staining.

### 2.3. Histopathological Analysis of Lung Tissue

The upper right lungs of mice were fixed with formalin and then embedded in paraffin. Four-micron-thick sections were prepared for staining with hematoxylin and eosin (HE). Histopathological analysis was performed by two pathologists blinded to the grouping under a light microscope with magnifications of 200x and 400x (Olympus, Tokyo, Japan). The histological alterations were graded based on an assessment of congestion, edema, inflammation, hemorrhage, and hyaline membrane formation (0, minimum damage; 1, mild damage; 2, moderate damage; 3, severe damage; and 4, intense damage), according to a previous report [27].

### 2.4. Myeloperoxidase (MPO) Activity Detection

The upper left lung tissue was homogenized to prepare the 5% tissue homogenate. The MPO activity of lung tissue was measured using an MPO assay kit (Nanjing Jiancheng Bio-Engineering Institute, China) according to our previous report [16]. The MPO activity of each sample was normalized to the corresponding protein concentration.

### 2.5. Pulmonary Alveolocapillary Permeability

Pulmonary alveolocapillary permeability of mice was evaluated based on the total protein concentration in the BALF and the wet/dry weight (W/D) ratio according to our previous study [16]. The total protein concentration in the BALF was measured with a bicinchoninic acid (BCA) kit (Thermo Fisher Scientific, Waltham, MA, USA). The W/D ratio was calculated as the ratio of the wet weight to the dry weight. The whole lung was weighed immediately after removal (wet weight). The lungs were then dehydrated at 80°C for 48 h and reweighed (dry weight).

### 2.6. RNA Isolation and Quantitative Real-Time Polymerase Chain Reaction (PCR)

Total RNA was isolated from the lower left lung tissue, and real-time PCR was performed according to our previous study [28]. Briefly, 1 *μ*g total RNA was used to reverse transcribe into cDNA with a PrimeScript RT Reagent Kit with gDNA Eraser (TaKaRa Clontech, Kusatsu, Japan). Real-time PCR was conducted using the SYBR Premix Ex Taq II system (TaKaRa Clontech) on a deep-well real-time PCR detection system (CFX96 Touch; Bio-Rad, Hercules, CA, USA). The data were analyzed using the 2^-ΔΔCT^ method. Primer sequences of the mouse genes used in this study are shown in Table 1.

### 2.7. Survival Analysis

According to our previous study [25], mice were given a lethal dose of O111:B4 LPS from *Escherichia coli* (40 mg/kg, intraperitoneal) to induce ALI. Sixty C57BL/6 mice were divided randomly into three groups: control, ALI, and budesonide + ALI groups (*n* = 20 per group). Budesonide was administered at a dose of 0.5 mg/kg 1 h prior to the LPS injection. All mice were observed every 6 h for 72 h.

### 2.8. Macrophage Culture and Treatment

RAW 264.7 murine macrophages were cultured in Dulbecco's modified Eagle's medium (Gibco, Grand Island, NY, USA) containing 10% fetal bovine serum (HyClone, USA). Cells were seeded in 6-well culture plates at a density of 1 × 10^6^ cells/well. After overnight incubation, cells were divided into three groups: control, LPS + adenosine triphosphate (ATP), and budesonide + LPS + ATP groups. In the LPS + ATP group, cells were stimulated with LPS (100 ng/mL) for 135 min, followed by treatment with ATP (5 mM; Sigma-Aldrich) for an additional 45 min to activate the NLRP3 inflammasome, according to a previous report [29]. In the budesonide treatment group, budesonide was added 30 min prior to LPS stimulation.

### 2.9. Western Blot Analysis

Lung samples and cells were homogenized in cell lysis buffer for western blotting. The protein concentrations were quantified with a BCA kit (Thermo Fisher Scientific). Equal amounts of protein (30 *μ*g) were loaded, separated by sodium dodecyl sulfate polyacrylamide gel electrophoresis on 10% gels, and transferred to polyvinylidene difluoride membranes (Millipore, Burlington, MA, USA). The membranes were blocked with 5% fat-free milk for 2 h at room temperature and then incubated with specific primary antibodies (anti-NLRP3, 1 : 2000 (Cell Signaling Technology, Danvers, MA, USA); anti-caspase-1 p20, 1 : 1000 (R&D, Minneapolis, MN, USA)) at 4°C overnight. After washing three times, the membranes were incubated with peroxidase-conjugated secondary antibodies (1 : 5000, Cell Signaling Technology) at room temperature for 1 h. Subsequently, bands were visualized with chemiluminescence (Millipore). The intensity of each band was analyzed using ImageJ (NIH, USA).

### 2.10. Enzyme-Linked Immunosorbent Assay (ELISA)

The concentrations of cytokines (tumor necrosis factor-*α* (TNF-*α*), IL-8, monocyte chemoattractant protein-1 (MCP-1), IL-1*β*, and IL-18) in BALF and IL-1*β* in the cell culture supernatant were measured by ELISA according to the manufacturer's instructions. ELISA kits for TNF-*α*, IL-8, and MCP-1 were purchased from Invitrogen (Thermo Fisher Scientific). The ELISA kits for IL-1*β* and IL-18 were purchased from BioLegend (San Diego, CA, USA).

### 2.11. Statistical Analysis

Data were expressed as means ± standard deviations. Data were analyzed using SPSS 17.0 (IBM Co., Chicago, IL, USA). Statistical analysis among means was performed using analysis of variance, followed by Turkey's post hoc test. Survival rates were evaluated by the *Kaplan-Meier* test. Differences with *P* values of less than 0.05 were considered statistically significant.

## 3. Results

### 3.1. Budesonide Attenuated Pathological Injury Induced by LPS in the Lungs of Mice

**Figure 1:**
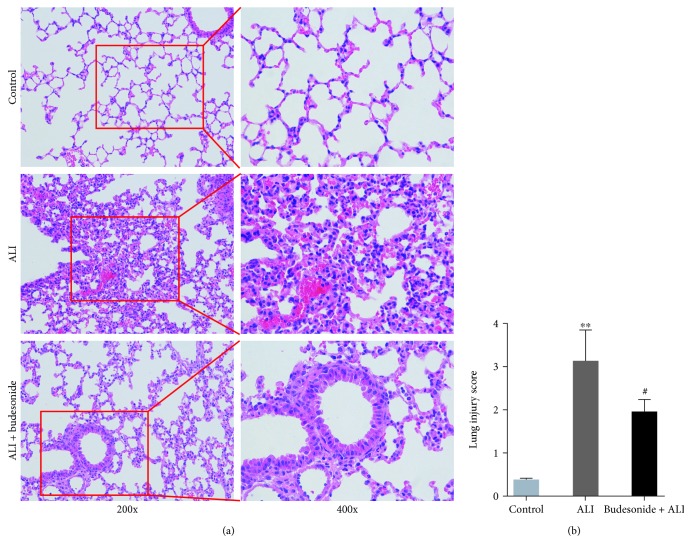
Budesonide attenuated the histopathological injury induced by LPS in mice. C57BL/6 mice received LPS administration (5 mg/kg, *i.t.*) with/without budesonide pretreatment (0.5 mg/kg). Twelve hours after LPS injection, histopathological changes were observed using HE staining (a). Lung injury scores were used to semiquantitatively evaluate the histopathological injury (b). Data were expressed as means ± standard deviations (*n* = 8). ^∗∗^*P* < 0.01 compared with the control group and ^#^*P* < 0.05 compared with the ALI group.

First, histopathological changes in lung tissues were evaluated by HE staining. We found that LPS administration (5 mg/kg, *i.t.*) resulted in obvious histopathological injury, including edema, accumulation of proinflammatory cells, and thickening of the alveolar wall, at 12 h after injection (Figure 1(a)). Budesonide attenuated these histopathological changes induced by LPS (Figure 1(a)). Similar findings were observed for lung injury scores (Figure 1(b), *P* < 0.05). These results indicated that budesonide attenuated pathological injury induced by LPS in the lung.

### 3.2. Budesonide Alleviated Pulmonary Microvascular Permeability in LPS-Treated Mice

**Figure 2:**
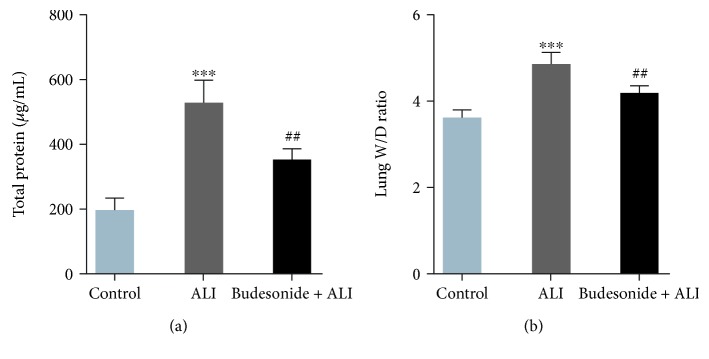
Budesonide attenuated lung edema. C57BL/6 mice were administered with LPS (5 mg/kg, *i.t.*) with/without budesonide pretreatment (0.5 mg/kg). Twelve hours after the LPS injection, the BALF was collected, and total protein concentrations were detected with a BCA kit (a). Lungs were collected for W/D ratio detection (b). Data were expressed as means ± standard deviations (*n* = 8). ^∗∗∗^*P* < 0.001 compared with the control group and ^##^*P* < 0.01 compared with the ALI group.

Edema is a typical pathological characteristic of the lungs with ALI. Both the increased total protein concentration in the BALF and the elevated W/D ratio indicated edema and impaired pulmonary microvascular permeability in LPS-challenged mice (Figure 2, *P* < 0.001). In contrast, pretreatment with budesonide (0.5 mg/kg) markedly decreased the total protein concentration in BALF and the W/D ratio compared with that in the ALI group (Figure 2, *P* < 0.001). These data suggested that budesonide alleviated the pulmonary microvascular permeability observed in LPS-treated mice.

### 3.3. Budesonide Inhibited the Infiltration of Proinflammatory Cells in the Lungs of LPS-Treated Mice

**Figure 3:**
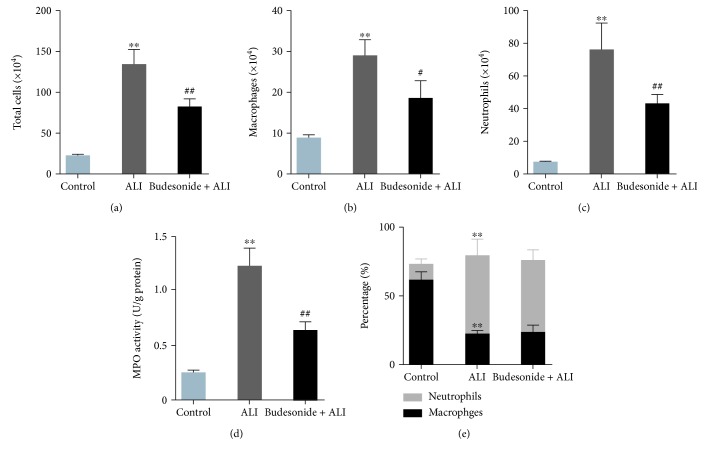
Budesonide inhibited the infiltration of proinflammatory cells induced by LPS in mice. Twelve hours after the administration of LPS (5 mg/kg, *i.t.*), BALF was collected and the numbers of cells were counted, including total cells (a), macrophages (b), and neutrophils (c). The activity of MPO in lung tissue was detected to reflect neutrophil recruitment (d). The percentage of neutrophil and macrophages in the BALF (e). Data were expressed as means ± standard deviations (*n* = 8). ^∗∗^*P* < 0.01 compared with the control group, ^#^*P* < 0.05 compared with the ALI group, and ^##^*P* < 0.01 compared with the ALI group.

Next, the infiltration of proinflammatory cells, including macrophages and neutrophils, was evaluated. Instillation of LPS (5 mg/kg) for 12 h resulted in increased BALF cell numbers, including macrophages and neutrophils (Figures 3(a)–3(c), *P* < 0.01). The administration of budesonide (0.5 mg/kg) dramatically suppressed the infiltration of proinflammatory cells in the BALF (Figures 3(a)–3(c), *P* < 0.05). The results of MPO activity reconfirmed that budesonide inhibited the infiltration of neutrophils induced by LPS in mice (Figure 3(d), *P* < 0.01). Additionally, neutrophils were the predominant proinflammatory cells during the progression of ALI in mice. However, the relative proportion of neutrophils and macrophages was not different in the budesonide-treated group (Figure 3(e), *P* < 0.01). These findings indicated that budesonide treatment suppressed the infiltration of proinflammatory cells induced by LPS in the lungs.

### 3.4. Budesonide Negatively Regulated LPS-Induced Production of Inflammatory Mediators in the Lung

**Figure 4:**
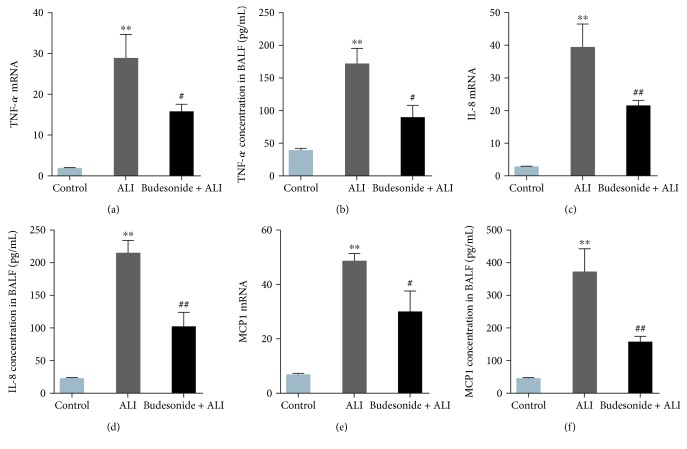
Budesonide suppressed the production of proinflammatory mediators induced by LPS. Twelve hours after LPS administration, the mRNA expression levels of *TNF-α* (a), *IL-8*, (c) and *MCP-1* (e) in the lung were detected by real-time PCR. The protein concentrations of TNF-*α* (b), IL-8 (d), and MCP-1 (f) in the BALF were detected by ELISA. Data were expressed as means ± standard deviations (*n* = 8). ^∗∗^*P* < 0.01 compared with the control group, ^#^*P* < 0.05 compared with the ALI group, and ^##^*P* < 0.01 compared with the ALI group.

Previous studies have demonstrated that LPS increases the levels of inflammatory mediators, including TNF-*α*, IL-8, and MCP-1 [16, 30, 31]. We also found that mRNA expression in lung tissue and protein expression of TNF-*α*, IL-8, and MCP-1 in the BALF were significantly increased in the ALI group (Figure 4, *P* < 0.01). Budesonide treatment resulted in significant decreases in these inflammatory mediators compared with the ALI group (Figure 4, *P* < 0.05). These data indicated that budesonide negatively regulated the expression of inflammatory mediators in LPS-induced lungs.

### 3.5. Budesonide Improved the Survival of LPS-Treated Mice

**Figure 5:**
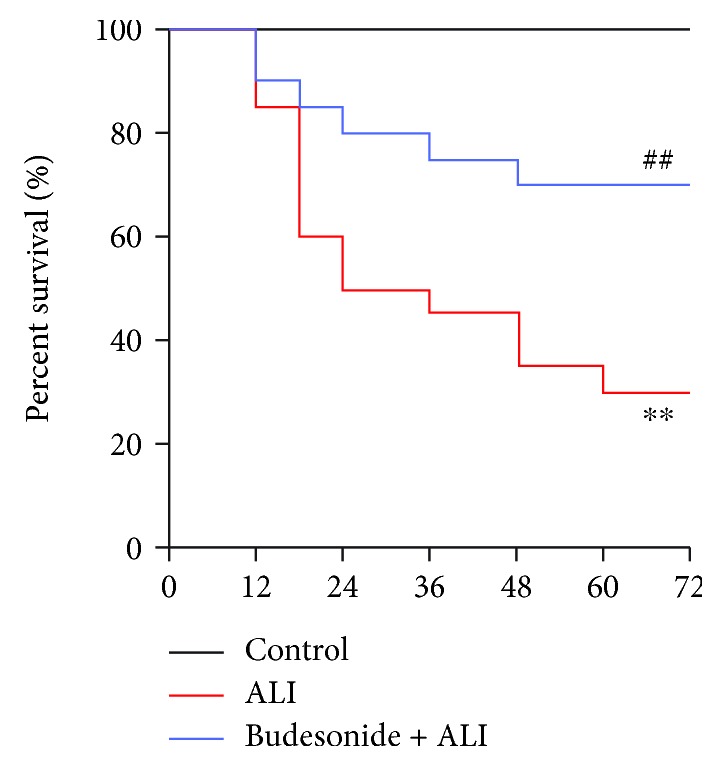
Budesonide attenuated LPS-induced mortality. C57BL/6 mice received a lethal dose of LPS (40 mg/kg, *i.t.*). The mortality of the animals was monitored every 6 h after LPS administration (*n* = 20). ^∗∗^*P* < 0.01 compared with the control group and ^##^*P* < 0.01 compared with the ALI group.

To assess the effects of budesonide on the survival of mice with ALI, a lethal dose of LPS (40 mg/kg) was injected (*i.t.*), which resulted in about 70% death within 72 h (Figure 5, *P* < 0.01). The mortality rate dropped to 25% in the budesonide-treated group (Figure 5, *P* < 0.01). These findings suggested that budesonide improved the survival of ALI mice.

### 3.6. Budesonide Inhibited the Activation of the NLRP3 Inflammasome Induced by LPS in Mice

**Figure 6:**
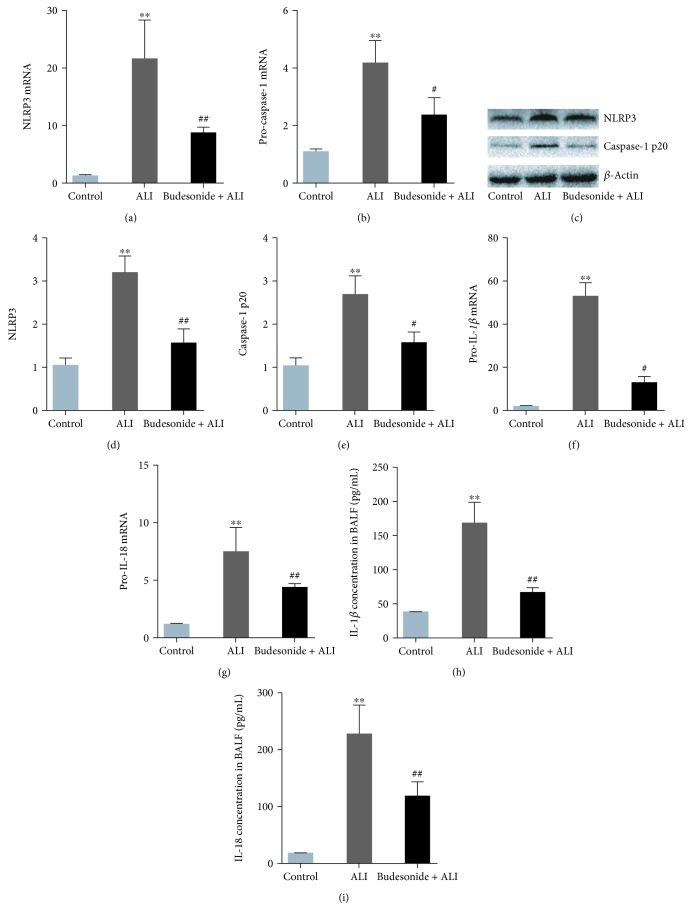
Budesonide pretreatment suppressed NLRP3 inflammasome activation in the lungs of LPS-treated mice. Twelve hours after LPS administration, the mRNA expression levels of *NLRP3* (a), *pro-caspase-1* (b), *pro-IL-1β* (f), and *pro-IL-18* (g) were detected using real-time PCR. The protein expression of levels NLRP3 (c, d) and caspase-1 p20 (c, e) were detected using western blotting. The concentrations of IL-1*β* (h) and IL-18 (i) in the BALF were detected using ELISA. Data were expressed as means ± standard deviations (*n* = 8). ^∗∗^*P* < 0.01 compared with the control group, ^#^*P* < 0.05 compared with the ALI group, and ^##^*P* < 0.01 compared with the ALI group.

The results showed that the components of the NLRP3 inflammasome, including NLRP3, pro-caspase-1, pro-IL-1*β*, and pro-IL-18, were significantly increased in the lungs of mice with LPS-induced ALI (Figures 6(a)–6(g), *P* < 0.01). Budesonide pretreatment partially restored these effects, indicating inhibition of the NLRP3 inflammasome (Figures 6(a)–6(g), *P* < 0.05). Additionally, budesonide also dramatically reduced NLRP3 inflammasome activation by modulating the expression of caspase-1 p20 and the secretion of IL-1*β* and IL-18 (Figures 6(c), 6(e), and 6(h)–6(i); *P* < 0.05). Taken together, these data indicated that inhibition of NLRP3 inflammasome activation contributed to the protective effects of budesonide against LPS-induced ALI in mice.

### 3.7. Budesonide Suppressed the Activation of the NLRP3 Inflammasome in Macrophages *In Vitro*

**Figure 7:**
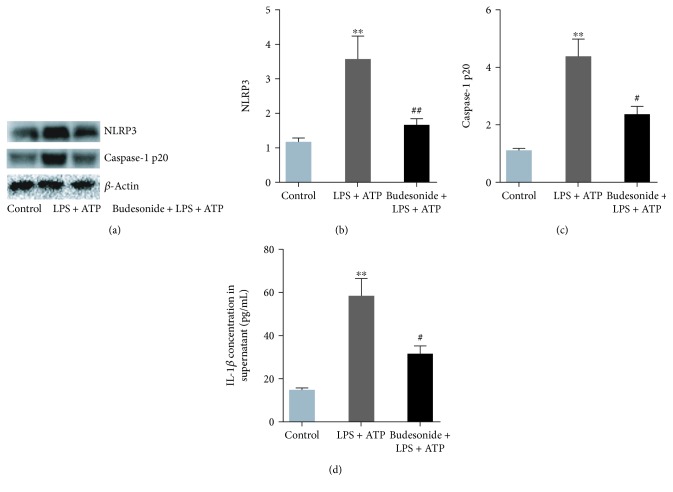
Budesonide suppressed NLRP3 inflammasome activation in macrophages *in vitro*. RAW 264.7 cells were treated with LPS (100 ng/mL) + ATP (5 mM) with or without budesonide. The protein expression levels of NLRP3 (a, b) and caspase-1 p20 (a, c) were detected using western blotting. The concentrations of IL-1*β* in the supernatant were detected using ELISA (d). Data were expressed as means ± standard deviations (*n* = 3). ^∗∗^*P* < 0.01 compared with the control group, ^#^*P* < 0.05 compared with the LPS + ATP group, and ^##^*P* < 0.01 compared with the LPS + ATP group.

To elucidate the effects of budesonide on the activation of the NLRP3 inflammasome, RAW 264.7 cells were used *in vitro*. Upon activation by LPS (100 ng/mL) plus ATP (5 mM), the levels of NLRP3, caspase-1 p20, and secreted IL-1*β* were increased (Figure 7, *P* < 0.01). Budesonide pretreatment reduced NLRP3 inflammasome activation (Figure 7, *P* < 0.05). Collectively, these data suggested that budesonide suppressed the activation of the NLRP3 inflammasome in macrophages *in vitro*.

## 4. Discussion

Although budesonide has been shown to have therapeutic effects in ALI/ARDS in some animal models [8–11], the exact effects of budesonide on LPS-induced ALI and the molecular mechanisms involved in these effects are still poorly understood. In our present study, we demonstrated, for the first time, that budesonide inhalation attenuated LPS-induced ALI in mice, as supported by reduced lung injury, suppressed production of proinflammatory cytokines, and improved survival rates. We showed that budesonide inhibited the activation of the NLRP3 inflammasome in lung tissues *in vivo* and in macrophages *in vitro*.

The mechanism through which budesonide attenuated ALI in mice is not entirely clear. In a lung injury model in rabbits induced by LPS injection via the ear vein [9], budesonide inhalation ameliorated lung injury by suppressing the production of proinflammatory cytokines. However, the mechanisms through which budesonide suppressed the inflammatory reaction were not evaluated. In another study, Ju and colleagues [22] demonstrated that budesonide inhibited the activation of NF-*κ*B. Our previous study showed that excessive NLRP3 inflammasome activation is the critical factor during ALI [16]. Moreover, in this study, we found that budesonide attenuated LPS-induced ALI in mice by suppressing NLRP3 inflammasome activation, providing a more specific mechanism. Accumulating evidence has demonstrated that excessive activation of the NLRP3 inflammasome mediates lung injury induced by infection [32], hyperoxia [33], and mechanical ventilation [34]. Based on the critical role of the NLRP3 inflammasome during ALI and our findings in this study, we suggest that the NLRP3 inflammasome may be an important target of budesonide.

To the best of our knowledge, this is the first report indicating that budesonide inhibited the activation of the NLRP3 inflammasome in lung tissue and macrophages. However, other glucocorticoids, such as prednisone, exert inhibitory effects on the NLRP3 inflammasome [20]. Interestingly, some studies have indicated that glucocorticoids activate the NLRP3 inflammasome under specific given conditions. For example, prenatal administration of dexamethasone, a synthetic glucocorticoid, increases the expression of inflammasome components (NLRP3 and caspase-1) in hippocampal oligodendrocytes of offspring in mice [35]. In addition, activation of the NLRP3 inflammasome causes glucocorticoid resistance in leukemia cells by cleavage of the glucocorticoid receptor [36] and decreased promoter methylation of caspase-1 and NLRP3 [37]. These reports indicate a strong cross-talk between the NLRP3 inflammasome and glucocorticoids. Nevertheless, the present findings confirmed that budesonide negatively controlled the activation of the NLRP3 inflammasome in LPS-induced ALI.

Activation of the NLRP3 inflammasome requires two independent signals [38]. The first is an initial priming signal, which induced transcriptional upregulation of NLRP3, caspase-1, and pro-IL-1*β* mainly *via* NF-*κ*B signaling [39]. Budesonide inhibits the activation of NF-*κ*B [22], contributing to the inhibitory effects of budesonide on NLRP3 inflammasome priming. The second stimulus is required to activate NLRP3 to induce inflammasome assembly [40]. Various danger signals, including mitochondrial reactive oxygen species, potassium efflux, and the release of lysosomal cathepsin, are possible activators of the NLRP3 inflammasome [41]. Although we found that budesonide treatment reduced the production of NLRP3 inflammasome activation *in vivo* and *in vitro*, e.g., by enhancing caspase-1 p20 expression and IL-1*β* and IL-18 secretion, the mechanism is still unknown; thus, further studies are needed.

Consistent with previous studies, other mechanisms are involved in the protective effects of glucocorticoids in ALI. For example, methylprednisolone ameliorates ALI induced by LPS (*i.v.*) by promoting macrophage M2 polarization [42], which enhances the resolution of experimental ARDS [43]. Based on analysis of individual patient data from four randomized trials, prolonged methylprednisolone treatment accelerates the resolution of ARDS, decreases hospital stay and healthcare utilization, and improves a broad spectrum of interrelated clinical outcomes [44]. The key contribution of this work is the finding that glucocorticoid (such as budesonide) treatment can attenuate lung injury induced by LPS partially through inhibition of NLRP3 inflammasome activation.

One limitation in our study was that budesonide treatment was performed before induction of ALI, which is contrary to the standard approach in clinical medicine. However, our data at least, partially revealed the protective effects of budesonide against ALI induced by LPS. Second, the dose of budesonide given in this study was 0.5 mg/kg, which was higher than the dose of inhaled budesonide used in clinical practice [45]. The dose of budesonide was chosen based on our preliminary experimental data and other reports [23]. The higher dose of budesonide may be necessary owing to the intranasal delivery and species variations. Intratracheal delivery may be more direct. Thus, in our future studies, we will investigate the therapeutic effects of budesonide for ALI at different times and doses.

In conclusion, we demonstrated that budesonide ameliorates lung injury and suppresses uncontrolled inflammation *via* inhibition of the NLRP3 inflammasome in LPS-induced ALI, indicating that budesonide inhalation may be an effective therapy for ARDS in clinical practice.

## Figures and Tables

**Table 1:** Primer sequences used to quantify gene expression in this study.

Gene	Forward primer (5′ to 3′)	Reverse primer (5′ to 3′)
*TNF-α*	CCACCCGCTCTTCTGTCTA	TGGTTTGTGAGTGAGGGT
*MCP-1*	GTCCCTGTCATGCTTCTGG	GCGTTAACTGCATCTGGCT
*IL-8*	ATGCCTCTCCATTTCCTGCT	CATGGGGAAAGAGGCTCTGA
*NLRP3*	TACGGCCGTCTACGTCTTCT	CGCAGATCACACTCCTCAAA
*Pro-caspase-1*	CACAGCTCTGGAGATGGTGA	CAGGCAGGCAGTATCACTCA
*Pro-IL-1β*	CAGGCAGGCAGTATCACTCA	AGCTCATATGGGTCCGACAG
*Pro-IL-18*	ACGTGTTCCAGGACACAACA	CAAACCCTCCCCACCTAACT
*β-Actin*	CACCATGTACCCAGGCATTG	CCTGCTTGCTGATCCACATC

## Data Availability

The data used to support the findings of this study are available from the corresponding author upon request.
